# ZAP70, too little, too much can lead to autoimmunity*

**DOI:** 10.1111/imr.13058

**Published:** 2021-12-18

**Authors:** Judith F. Ashouri, Wan‐Lin Lo, Trang T. T. Nguyen, Lin Shen, Arthur Weiss

**Affiliations:** ^1^ Department of Medicine Rosalind Russell and Ephraim P. Engleman Rheumatology Research Center University of California, San Francisco San Francisco California USA; ^2^ Division of Microbiology and Immunology Department of Pathology University of Utah Salt Lake City Utah USA; ^3^ Howard Hughes Medical Institute University of California, San Francisco San Francisco California USA

**Keywords:** autoimmunity, LAT, TCR signaling, tolerance and anergy, ZAP70

## Abstract

Establishing both central and peripheral tolerance requires the appropriate TCR signaling strength to discriminate self‐ from agonist‐peptide bound to self MHC molecules. ZAP70, a cytoplasmic tyrosine kinase, directly interacts with the TCR complex and plays a central and requisite role in TCR signaling in both thymocytes and peripheral T cells. By studying *ZAP70* hypomorphic mutations in mice and humans with a spectrum of hypoactive or hyperactive activities, we have gained insights into mechanisms of central and peripheral tolerance. Interestingly, both hypoactive and hyperactive ZAP70 can lead to the development of autoimmune diseases, albeit through distinct mechanisms. Immature thymocytes and mature T cells rely on normal ZAP70 function to complete their development in the thymus and to modulate T cell responses in the periphery. Hypoactive ZAP70 function compromises key developmental checkpoints required to establish central tolerance, allowing thymocytes with potentially self‐reactive TCRs a greater chance to escape negative selection. Such ‘forbidden clones’ may escape into the periphery and may pose a greater risk for autoimmune disease development since they may not engage negative regulatory mechanisms as effectively. Hyperactive ZAP70 enhances thymic negative selection but some thymocytes will, nonetheless, escape negative selection and have greater sensitivity to weak and self‐ligands. Such cells must be controlled by mechanisms involved in anergy, expansion of Tregs, and upregulation of inhibitory receptors or signaling molecules. However, such potentially autoreactive cells may still be able to escape control by peripheral negative regulatory constraints. Consistent with findings in *Zap70* mutants, the signaling defects in at least one ZAP70 substrate, LAT, can also lead to autoimmune disease. By dissecting the similarities and differences among mouse models of patient disease or mutations in ZAP70 that affect TCR signaling strength, we have gained insights into how perturbed ZAP70 function can lead to autoimmunity. Because of our work and that of others on ZAP70, it is likely that perturbations in other molecules affecting TCR signaling strength will be identified that also overcome tolerance mechanisms and cause autoimmunity. Delineating these molecular pathways could lead to the development of much needed new therapeutic targets in these complex diseases.

## 1 INTRODUCTION

**FIGURE 1:**
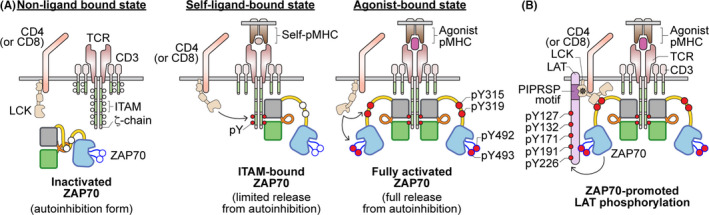
Illustration of ZAP70 activities during TCR recognition of self‐pMHC or agonist‐pMHC. (A) In the resting state, when the TCR does not interact with any pMHC, ITAMs are not phosphorylated and ZAP70 remains in an autoinhibited state in the cytoplasm. When the TCR recognizes a self‐pMHC, the interaction with self‐pMHC delivers a sufficient tonic signal to lead to ITAM phosphorylation by the kinase LCK. This then results in the recruitment of ZAP70 to the phosphorylated ITAM motifs but ZAP70 does not become phosphorylated. The degree of self‐reactivity is associated with the amount of ζ‐chain phosphorylation. Upon recognition of agonist pMHC, coreceptor‐associated LCK is brought into the vicinity of engaged TCR and CD3 complexes long enough to phosphorylate ITAMs and recruit as well as phosphorylate ZAP70 that is bound to phosphorylated‐ITAMs. Phosphorylation of ZAP70 by LCK on Y315, Y319 and Y493 leads to its activation. (B) Activated ZAP70 subsequently phosphorylates LAT on five key tyrosine residues. Notably, the SH3 domain of LCK (marked with *) interacts with the PIPRSP motif of LAT to actively recruit LAT to the activated ZAP70. This active recruitment of LAT by LCK enhances ZAP70‐mediated LAT phosphorylation and augments T cell sensitivity towards weak ligand stimuli. Furthermore, phosphorylated LAT and another ZAP70 substrate, SLP76, can function as scaffold proteins to recruit other downstream signaling proteins and events, which can eventually lead to T cell activation

A healthy adaptive immune system not only requires a robust response to pathogens or to potential oncogenic mutations but also requires the absence of aggressive reactivity to self. This state of unresponsiveness to self generally defines a state of tolerance. Reactivity, of course, depends upon the recognition by the T cell antigen receptor (TCR) of pathogen‐ or oncogene‐derived peptides bound to class I or class II MHC molecules (agonist pMHC). Such recognition of agonist pMHC leads to generation of activation signals dependent upon well‐characterized intracellular biochemical events occurring at the TCR (Figure 1). These events generate intracellular downstream signals that contribute to T cell activation and that can lead to clonal T cell proliferation, differentiation, and acquisition of effector functions.

The robust response to antigenic threats by the host does not imply that there is a complete lack of any recognition of self‐pMHC since naive T cells depend upon recognition of self‐pMHC for survival.^1^ Self‐pMHC binding to the TCR generates weaker or qualitatively distinct “tonic” signals that ensure T cell survival.^2^, ^3^, ^4^ The affinity differences between an agonist‐pMHC (typically low μM range) vs a self‐pMHC with the TCR is remarkably small (10‐15‐fold differences).^5^ Yet, T cells are remarkably sensitive. A single pMHC is sufficient to initiate the response of some T cells and responses are generally very specific.^3^ Several different models for such ligand discrimination have been suggested to account for the TCR sensitivity and selectivity (reviewed in refs.^6^, ^7^). Ultimately, each model depends upon signal transduction events generated by the TCR. These signaling events play roles in all the mechanisms involved in preventing a robust response to self‐pMHC and establishing a state of self‐tolerance.

Distinct tolerance mechanisms operate centrally or peripherally. Central T cell tolerance occurs during thymocyte development. Cells expressing clonally rearranged and displayed TCRs undergo a quality control assessment to select for a population of thymocytes expressing TCRs that interact appropriately (positive selection) but not too strongly with self‐pMHC (reviewed in ref.^8^). Central tolerance depends upon negative selection which occurs by the elimination of thymocytes with TCRs that interact too strongly with self‐pMHC. Such strong reactivity generates potent TCR‐dependent signals that lead to apoptosis of these potentially autoreactive cells.

Negative selection is not perfect.^9^ Some potentially autoreactive cells escape into the periphery because signals in the thymus were not strong enough to induce apoptosis or the autoantigen might not have been sufficiently encountered. Several mechanisms account for how autoreactivity is normally prevented in the periphery: induction of T cell anergy—an induced state of unresponsiveness^10^; suppression of responsiveness of antigen‐specific T cells by T regulatory cells (Tregs)^11^; or by the effects of inhibitory signaling mechanisms.^12^, ^13^ Naive T cells normally require signals delivered via their TCR and via a co‐stimulatory receptor, typically CD28, to become productively activated.^14^ CD80 and CD86, are induced on antigen‐presenting cells by activation of innate immunity by pathogens or perhaps via inflammation induced by a tumor. Stimulation of CD28 ensures that naive T cells are not induced in the absence of inflammation, that is, so that T cells are not responding to a self‐antigen encountered outside of the thymus. In the presence of a strong TCR signal, but in the absence of a co‐stimulatory signal, T cells enter a state of unresponsiveness called anergy. Another mechanism to prevent activation of potentially autoreactive cells is via the suppressive influence of Tregs, which may share some level of specificity with stimulated T cells. Finally, sufficient induction of TCR signaling, acutely or chronically, may induce adaptive mechanisms to suppress TCR signaling via ubiquitin ligases or inhibitory receptors. This may be a means to prevent immunopathology during chronic infections.

Discrimination of ligands and induction of central and peripheral tolerance mechanisms depends upon signal transduction by the TCR. Appropriate signaling strength or quality is required for negative selection, anergy induction, Treg generation and activity, as well as induction of inhibitory feedback mechanisms (ie, E3 ubiquitin ligases or inhibitory receptors). Here, we will focus on several systems in which inappropriate or insufficient signaling via the TCR alters the tolerance threshold centrally or peripherally. We will focus our discussion on recent work from our labs that have studied mutants of a single kinase, ZAP70, and its substrate, LAT. ZAP70 is a cytoplasmic tyrosine kinase critically important in proximal events involved in TCR signaling. The mutants we will discuss either decrease or increase TCR signaling function.

### 1.1 The role and regulation of ZAP70 in TCR signaling

Together with LCK, ZAP70 plays a critical role in proximal events linked to initiating TCR signaling. Engagement of the TCR by pMHC or by anti‐receptor (anti‐TCRαβ or anti‐CD3) monoclonal antibodies (mAbs) initiates critical biochemical events involving LCK and ZAP70 that are summarized for simplicity in Figure 1. ZAP70 plays a critical requisite role. In the absence of ZAP70, distal signaling events leading to calcium increase, Ras and PKC activation, actin cytoskeletal remodeling, and various transcriptional responses do not occur.^15^ The consequence of loss of ZAP70 function is immunodeficiency in both mouse and man.^16^

LCK, like other SRC family kinases (SFKs), can autoactivate via trans‐autophosphorylation. Thus, in the resting T cell, a pool of active LCK exists.^17^ This active pool of LCK is established via the combined actions of the inhibitory cytoplasmic tyrosine kinase CSK which phosphorylates Y505 in the C‐terminal segment of LCK and the positive regulatory control of the CD45 tyrosine phosphatase which dephosphorylates phospho‐Y505.^6^ A basal homeostatic state in which a relatively stable amount of LCK is active is established through the combined actions of CSK and CD45.^18^ Fluctuations are presumably controlled through feedback mechanisms that control CSK localization to the plasma membrane via adaptors that serve as substrates for SFKs in order to regulate the amount of CSK at the plasma membrane and thereby negatively regulate LCK activity.

In the non‐ligand‐bound state, the TCR CD3‐ and ζ‐chains are not tyrosine phosphorylated (see Figure 1, left panel). However, interaction of the TCR with appropriate self‐pMHC induces LCK‐mediated tyrosine phosphorylation of an evolutionarily conserved sequence motif termed ITAM (for immunoreceptor tyrosine‐based activation motif) expressed as a single copy in each of the CD3‐chains and is triplicated in the ζ‐chain.^19^ In the case of pMHC stimulation, the co‐engagement of CD4 or CD8 with pMHC contributes to the localization of LCK with the stimulated TCR, thereby facilitating ITAM phosphorylation.

ZAP70 and SYK are closely related kinases but are differentially expressed.^20^ ZAP70 replaces SYK during thymic development. SYK plays a more important role early in thymocyte development, specifically in TCR β‐selection, when co‐receptors are not involved and pre‐TCR expression at the plasma membrane is the main signal for progression of the TCR alpha‐beta lineage. SYK‐dependent signaling via the pre‐TCR is required for its own downregulation and the upregulation of ZAP70.^21^ Three features of ZAP70 distinguish it from SYK and likely account for the switch to ZAP70 during thymocyte development.^16^ First, ZAP70 is more tightly autoinhibited than SYK.^22^ Second, unlike SYK, ZAP70 cannot autoactivate but instead requires phosphorylation input from a SFK.^16^ Third, ZAP70 has a distinct set of substrates from SYK and from SFKs, whereas the substrate specificity of SYK and SFKs partially overlap.^23^ To a large extent these differences could reflect the establishment of a hierarchical relationship between LCK and ZAP70 to enforce CD4/CD8 participation in facilitating the TCR preferential recognition of pMHC.

**FIGURE 2:**
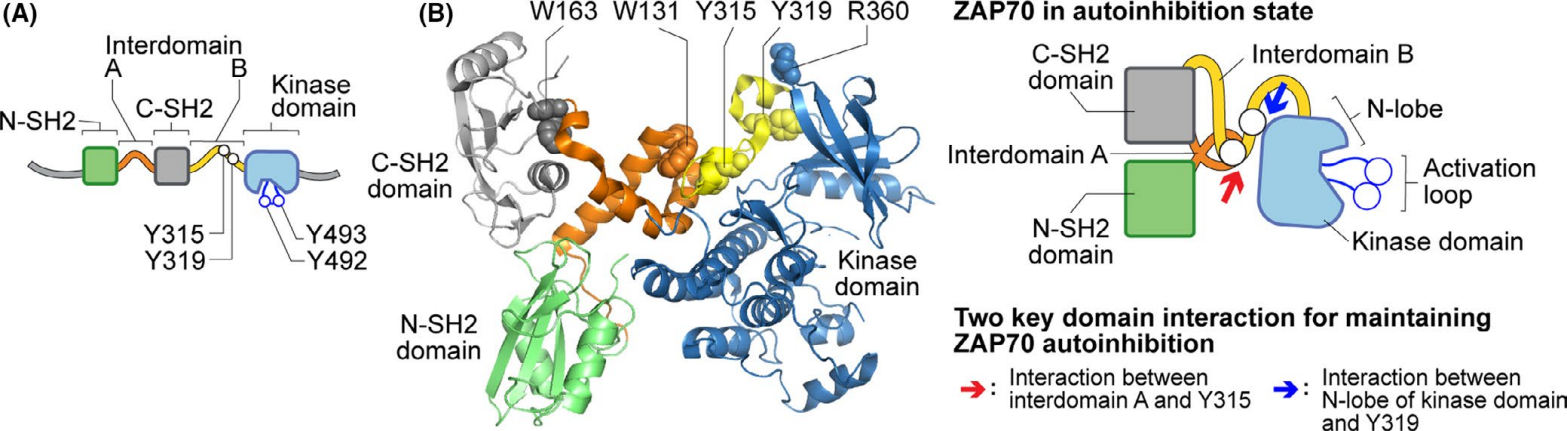
Illustration of ZAP70 structure and signaling domains. (A) Cartoon of ZAP70 structural domains, including the N‐terminal and C‐terminal SH2 domains, interdomain A and B, and a kinase domain. Some of the tyrosine residues that are phosphorylated by LCK are also shown (red open circles). (B) A crystal structural model (left) or a schematic (right) of autoinhibited ZAP70. The key amino acid residues (illustrated by spheres) at which the mutations in ZAP70 described in this review are also labeled (W131, W163, Y315, Y319 and R360). The red and blue arrows indicate the key interaction among different structural domains of ZAP70 to maintain ZAP70 in the autoinhibitory state

Although membrane bound and co‐receptor‐associated LCK is involved in the initiation of TCR signaling, ZAP70 normally resides in the cytoplasm in an autoinhibited conformation (Figure 2). A structural model of autoinhibited ZAP70 is depicted in Figure 2. If both tyrosines in an ITAM are phosphorylated by LCK, the tandem SH2 domains of ZAP70 bind to the doubly phosphorylated ITAM with high affinity (low nanomolar) and a high degree of specificity.^24^ The binding of the tandem SH2 domains to the doubly phosphorylated ITAM causes a conformational change in the inter‐SH2 domain (interdomain A) that partially relieves the autoinhibitory conformation.^16^ This appears to be the extent to which the initial events involving ZAP70 occur under the influence of self‐pMHC since ZAP70 is not phosphorylated under the influence of self‐pMHC. Indeed, in freshly isolated thymocytes or T cells from secondary lymphoid tissues, the ζ‐chains are tyrosine phosphorylated and non‐phosphorylated ZAP70 bound to the doubly phosphorylated ITAMs can be detected.^25^ The recruitment and limited relief of autoinhibition likely does not proceed further because pMHC is not bound to the TCR long enough to allow for LCK‐mediated phosphorylation of key residues in ZAP70 that are required for the further relief of autoinhibition and subsequent kinase activation.

The longer interaction that occurs when the TCR is bound to an agonist pMHC is accompanied by a more prolonged interaction with a CD4‐ or CD8‐coreceptor that may or may not have an active LCK molecule bound. This differential binding of active LCK with co‐receptors has been reported in recent work that suggests a co‐receptor scanning model may be involved in ligand discrimination.^26^ Longer duration of binding of co‐receptor‐associated active LCK with the agonist pMHC bound to the TCR is associated with phosphorylation of Y315 and Y319 in SH2‐kinase linker domain (interdomain B) and is also associated with phosphorylation of Y493 in the ZAP70 activation loop (Figure 1). The phosphorylation of Y493 by LCK, thereby stabilizing the kinase active conformation, is a critical step in activating ZAP70 catalytic function.^16^

The phosphorylation of Y315 and Y319 is also a critically important event. Phosphorylation of these sites stabilizes the open and active conformation of ZAP70 and also allows these phospho‐sites to interact with several other proteins including Vav, CrkL, and LCK. Interaction of Vav and CrkL couple ZAP70, and hence the stimulated TCR, to important actin cytoskeleton regulatory events.^16^ The interaction of phospho‐Y319 with the LCK SH2 domain is particularly notable, since it provides several positive feedback functions that serve to enhance ZAP70 activity and TCR signaling.^27^ First, it serves to maintain co‐receptor‐associated LCK near the stimulated TCR and active ZAP70, thereby promoting further phosphorylation of ITAMs and ZAP70 molecules. Second, the active conformation of LCK is maintained since the SH2 domain participates in autoinhibition of LCK. Third, the active conformation of ZAP70 is maintained, preventing ZAP70 from returning to an autoinhibited conformation. Fourth, the bridging of the SH2 domain of LCK to phospho‐Y319 of ZAP70 facilitates the localization of one of ZAP70's key substrates, LAT, via the SH3 domain of LCK (Figure 1B).^28^ Thus, the coordinated hierarchical interactions of LCK and ZAP70 serve to not only activate ZAP70 bound to a stimulated TCR but also serve to provide positive feedback functions to the ZAP70 molecule(s) bound to an agonist pMHC‐stimulated TCR.

### 1.2 ZAP70 substrate selectivity

The substrate specificity of ZAP70 is distinctly different from that of SYK and LCK. Most notable is the inability of ZAP70 to phosphorylate ITAMs, requiring it to depend upon LCK or other SFKs to initiate TCR signaling.^23^, ^29^ As mentioned, this substrate specificity conveniently couples LCK‐associated co‐receptor (CD4 or CD8) involvement to pMHC recognition. The inability of ZAP70 to autoactivate further couples ZAP70 dependency to co‐receptor‐associated LCK. On the other hand, co‐receptor signaling alone does not activate T cells. This is due to the distinct substrate selectivity of LCK and other SFKs, when compared to the substrates preferentially phosphorylated by active ZAP70. The most well‐characterized ZAP70 substrates are specific phospho‐sites in the adaptors LAT and SLP76.^16^, ^23^ These two adaptors are not phosphorylated by SFK family members.

The functions of LAT and SLP76 are both critical to T cell development and T cell function, as delineated in studies of cell lines and deficient mice. LAT is a transmembrane protein that functions, via five ZAP70‐mediated tyrosine phosphorylation sites, in the recruitment of effector molecules including PLCγ1 and GRB2/SOS to the plasma membrane in order to activate downstream pathways which lead to calcium, PKC, and Ras activation.^6^, ^30^, ^31^, ^32^, ^33^ SLP76, via three ZAP70‐mediated tyrosine phosphorylation sites and GADS (a GRB2‐related adaptor) coordinate the assembly of several signaling complexes including those that involve the TEC family kinase ITK that participates in PLCγ1 phosphorylation and activation.

The ZAP70‐mediated phosphorylation sites are notable for the relative absence of basic amino acids near the phosphorylated tyrosines and a relative enrichment of acidic residues, particularly at the −1 position relative to the phosphorylated tyrosine.^23^ These features are distinctly different from ITAM sequences which have multiple basic residues surrounding phospho‐tyrosines and do not have an acidic residue at the −1 position. The selectivity for the sites in LAT and SLP76 is explained by the high concentration of basic residues in the ZAP70 kinase domain substrate‐binding segment.^23^ These basic residues are highly conserved in ZAP70 sequences in more than 40 species sequences identified. Moreover, such a concentration of basic residues in the substrate‐binding segment are unusual in the other cytoplasmic tyrosine kinases. Among the 32 cytoplasmic tyrosine kinases, the kinase domain of ZAP70 is one of the three most basic and is distinguished from others since the basic residues are unusually concentrated in the ZAP70 kinase domain substrate‐binding region. Molecular dynamic simulations support the notion that the acidic substrate peptides dock with the basic residues in the ZAP70 catalytic domain.^23^ These findings suggest that the substrate‐binding region of ZAP70 serves as an electrostatic filter in finding its substrate tyrosines in LAT and in SLP76.

One feature of LAT that is noteworthy is a highly conserved proline‐rich region that plays a role in localizing LAT to the stimulated TCR complex (see Figure 1B).^28^, ^34^ We showed that this proline‐rich sequence in LAT interacts with the SH3 domain of LCK, presumably while activated LCK is bound to the stimulated TCR and to ZAP70 as discussed above.

It is clear that ZAP70 plays a critical role in TCR signaling. In previous studies, we have used a PP1 analog‐sensitive inhibitor model system to show that the catalytic activity is critically important for T cell responses in vitro and in thymocyte developmental transitions in thymic slices.^35^, ^36^ Titrating the degree of ZAP70 catalytic activity in a thymic slice system had impacts on the percentages of double positive thymocytes able to make developmental transitions. This suggests that strength of signaling has a direct influence on thymocyte selection, and likely on mature T cell responses. Here, we review recent studies from our laboratories that highlight the impact of well‐characterized human and mouse mutations on T cell development, mature T cell responses, and tolerance.

## 2 DECREASED ZAP70 FUNCTIONAL EFFECTS ON T CELL FUNCTION AND THE IMMUNE SYSTEM

### 2.1 ZAP70 loss of function leads to immune deficiency

It is well‐established that impaired TCR signaling leads to immune deficiency. Yet, ZAP70 deficiency in humans is a rare cause of combined immunodeficiency (CID) or severe CID (SCID) caused by recessive homozygous/compound heterozygous loss‐of‐function mutations in the *ZAP70* gene^37^, ^38^, ^39^ and as reviewed in^40^). Laboratory evaluation of patients is notable for a paucity of CD8 peripheral blood T cells and the presence of normal numbers of non‐functional CD4 T cells. Individuals with *ZAP70* mutations can present with varied clinical manifestations. Patients with ZAP70 deficiency are particularly susceptible to respiratory infections including viral, bacterial, fungal, and protozoal infections,^40^ and can also present with cutaneous disorders.^37^, ^40^, ^41^ Many of these manifestations have been comprehensively reviewed in a systematic review of 49 ZAP70‐deficient patients.^40^

### 2.2 A link between immunodeficiency and autoimmunity in humans

The link between immunodeficiency and autoimmunity has long been appreciated in humans. Perhaps it is most evident in patients with primary immunodeficiency where up to 20%‐25% may develop autoimmune cytopenias, rheumatoid arthritis (RA), or other autoimmune diseases.^42^, ^43^, ^44^ The greatest risk has been associated with partial T cell immunodeficiencies and common variable immunodeficiency.^44^ The reverse association has also been described. In the setting of primary autoimmunity, despite the presence of activated immune cells mediating disease, observational studies have found an increased susceptibility to serious infections (independent of immune suppressive medications) and premature aging of the immune system, suggesting that impaired immune function might be a primary defect that, in turn, subverts tolerance.^45^, ^46^, ^47^, ^48^ For example, RA CD4 T cells have the paradoxical ability to differentiate into pathogenic effector cells despite their impaired response to TCR engagement.^49^, ^50^, ^51^, ^52^, ^53^, ^54^, ^55^, ^56^, ^57^, ^58^ Importantly, this paradox is observed in the *Zap70* mutant SKG mouse model of arthritis (discussed below), making it one of the few mouse models to capture the contribution of an autoreactive TCR repertoire with impaired TCR signal transduction to the pathogenesis of arthritis.

Likewise, a subset of the ZAP70‐deficient patients described by Sharifinejad et al^40^ developed non‐infectious clinical manifestations, such as autoimmune disease (n = 7, 19.4%), including autoimmune cytopenias, autoimmune nephritis, and autoimmune enteropathy. The heterogeneity in *ZAP70* mutations, clinical manifestations, and frequent need for treatment with a hematopoietic stem cell transplant (HSCT), can make it difficult to achieve a comprehensive immunologic and molecular view of ZAP70 deficiency in humans. However, mouse models (eg, SKG mouse model) can provide additional insight into the importance of ZAP70 function for immune cell development, T cell repertoire selection, and the propensity for autoimmunity in *Zap70* murine mutants.

### 2.3 Impairments in TCR signaling can result in autoimmunity via disparate mechanisms

That T cells with severely impaired signaling can effectively differentiate into pathologic effector cells that mediate an inflammatory, autoimmune syndrome seems counter‐intuitive. Yet, there are multiple reports linking immune deficiency with immune dysregulation and autoimmunity.^52^, ^59^, ^60^, ^61^, ^62^, ^63^, ^64^, ^65^ It has been suggested that the partial reduction in the number or function of T cells can disturb a “tolerogenic balance” and thereby generate a combination of immunodeficiency and hyper‐immune dysregulation.^62^ The precise mechanism remains elusive, although murine models suggest multiple factors are at play, including selection of an autoimmune TCR repertoire, lymphopenia‐induced exaggerated homeostatic proliferation precipitating autoimmune disease, deficient Treg function, resistance to Treg suppression, and T cell dysregulation causing failed induction of anergy in autoreactive T cells despite chronic antigen stimulation.^60^, ^63^ Notably, ZAP70‐deficient mice, as well as LAT‐mutated mice (the latter discussed in more detail later in this review), reveal a causal link between impaired TCR signaling associated with immune dysregulation and autoimmunity.^66^, ^67^, ^68^, ^69^, ^70^ Similarly, mouse models with cytokine signaling defects such as in STAT5A/5B‐deficient mice can also serve as examples of immunodeficiency and immune dysregulation and suggest a synergistic role for cytokine signaling in the activation of self‐reactive T cells.^62^, ^71^

### 2.4 ZAP70 loss of function mutations can lead to autoimmunity in murine models

Impairments in T cell signaling are linked to the pathogenesis of certain T‐cell‐mediated autoimmune diseases in humans,^40^, ^72^ and are also described in murine models. Previously, Goodnow and colleagues identified two hypomorphic *Zap70* mutants from an ENU mutagenesis screen. Studies from these *Zap70* mutants demonstrated that impaired TCR signaling and thymic selection can produce a potentially autoreactive repertoire of TCRs, and revealed a signaling threshold below which autoimmunity develops.^62^, ^67^ Compound heterozygotes were generated and studied on the C57BL/6 genetic background in which these mice develop dsDNA autoantibodies, a characteristic of SLE, and hyper‐IgE syndrome.

**FIGURE 3:**
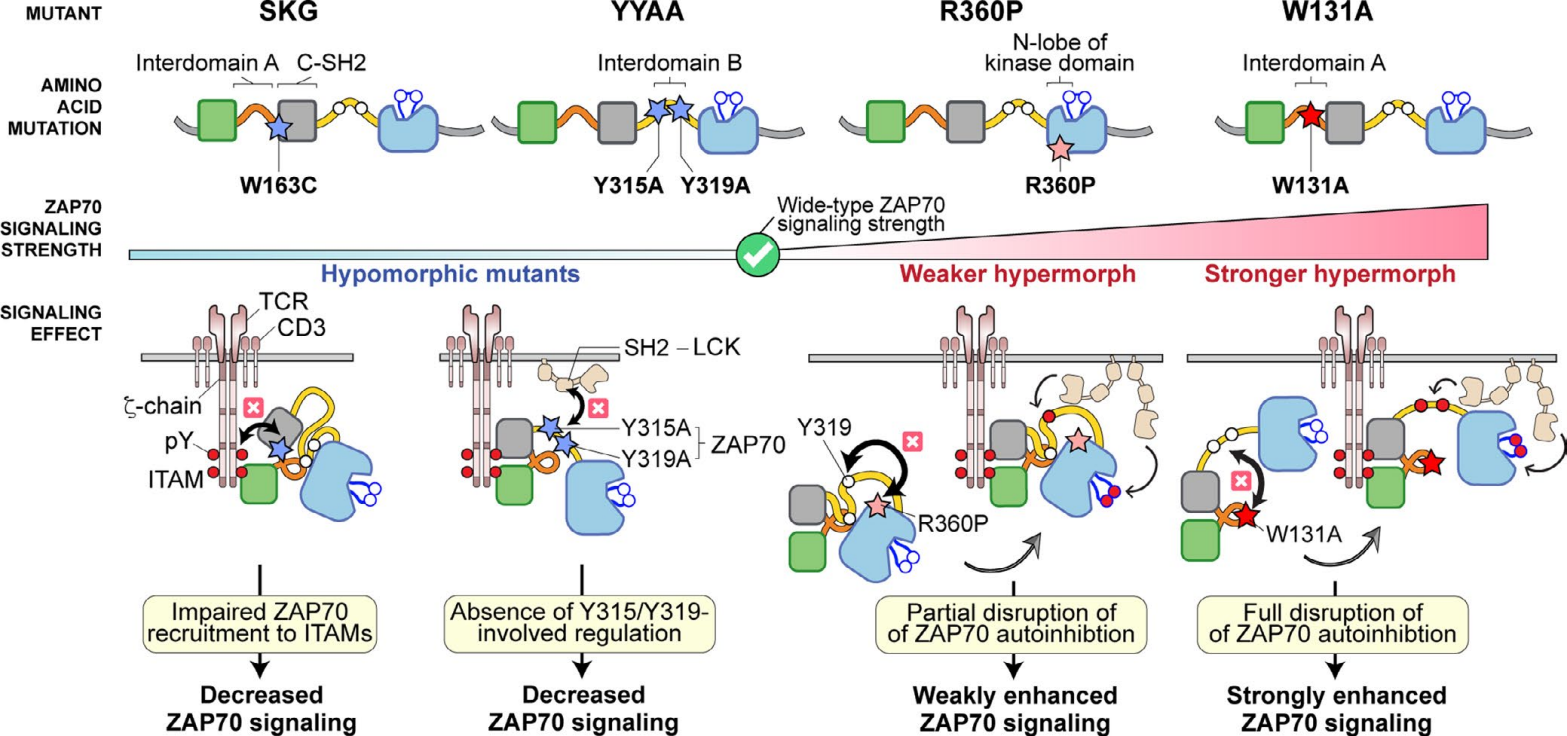
Allelic ZAP70 mutations directly impact T cell signaling strength. Hypomorphic and hypermorphic ZAP70 mutants and the resulting signaling defects are summarized. The stars represent the location of individual amino acid mutations: the blue stars represent hypomorphic mutants, the pink star represents weaker hypermorphic mutant and the red star represents the stronger hypermorphic mutant. All the signaling effects resulting from individual mutations are illustrated (black arrows and cross symbols). Red‐filled circles represent phosphorylated tyrosine residues

The SKG genetic mouse model of arthritis susceptibility on the BALB/c background, is yet another example of autoimmunity in the setting of immune deficiency.^64^ This may also reflect the importance of genetic background in driving specific manifestations of autoimmune disease, as these mice develop joint specific autoimmunity rather than features associated with SLE. The SKG mouse, first described in 2003, harbors a missense mutation (W163C) in ZAP70 (Figure 3).^64^ This mutated *Zap70* variant has a functionally impaired C‐SH2‐binding domain that prevents optimal interaction with the stimulated, phosphorylated TCR complex. This causes severely impaired downstream TCR signaling events resulting in altered positive and negative thymic selection. As a consequence, SKG T cells exhibiting the strongest affinity for self‐pMHC ligands are positively selected instead of undergoing deletion, giving rise to a more autoreactive TCR repertoire.^64^, ^65^, ^73^, ^74^ This allows for the escape of arthritogenic CD4 T cells, which are responsible for spontaneous T‐cell‐mediated arthritis, immunopathologically similar to human RA. Disease features include RA‐associated autoantibodies, destructive synovitis, and interstitial lung disease.^64^, ^75^ Studies of TCR transgenic mice crossed to the SKG mutation established that altered arthritogenic TCR specificity and altered TCR signaling are both essential for disease,^59^, ^64^, ^65^ and suggest that non‐specific T cell expansion in a lymphopenic host is not solely responsible for arthritis development in SKG mice. This highlights the importance of dysregulated peripheral tolerance due to altered TCR signaling in addition to TCR specificity in disease development.

The SKG mice not only exhibit a loss in central and likely peripheral tolerance that can be molecularly dissected but they also recapitulate the paradoxical ability of RA CD4 T cells to differentiate into pathogenic effector cells despite impaired TCR signaling.^49^, ^50^, ^51^, ^52^, ^53^, ^54^, ^55^, ^56^, ^57^, ^58^, ^64^, ^76^ In this model SKG CD4 T cells are sufficient and necessary to cause arthritis.^64^ We have shown that adoptive transfer of naive SKG CD4 T cells are sufficient to trigger disease.^76^

### 2.5 Impaired TCR signaling reveals a threshold for autoimmunity in the thymus

Our lab previously generated another *Zap70* mutant mouse (YYAA) in which two tyrosines, Y315 and Y319 (see Figure 3), are both mutated to alanine.^65^ The YYAA mutation resulted in an unanticipated partial loss of ZAP70 function—revealing the importance of the scaffolding roles of these two tyrosines. Like the SKG mouse, this mouse also has impaired T cell development, diminished TCR signaling (but not quite as severe), and defective negative and positive selection. These *Zap70* mutations were sufficient to drive rheumatoid factor (RF) production in BALB/c mice challenged with zymosan but did not produce frank arthritis in response to identical environmental challenges that produced arthritis in the SKG line.^65^ Direct comparison to SKG mice on the BALB/c background did not reveal substantial differences in IL‐17 production by T cells following zymosan treatment, and Treg function appears to be defective in both mice.^65^ The basis for the failure of this mouse to have a CD4 T cell response that leads to joint inflammation is not clear. We suspect that the SKG and YYAA mutants have differentially altered basal signaling (the homeostatic state of unstimulated T cells) and/or modification of their signaling circuitry (up or down regulation of signaling regulators) to alter the response potential in these two mice. Thus, these two *Zap70* mutations in YYAA and SKG mice may differentially skew their TCR repertoires and have T cells with altered TCR signaling in ways that manifest as different autoimmune syndromes.

### 2.6 Nur77‐TCR signaling reporter permits identification of arthritogenic CD4 T cells in RA‐prone SKG mice

**FIGURE 4:**
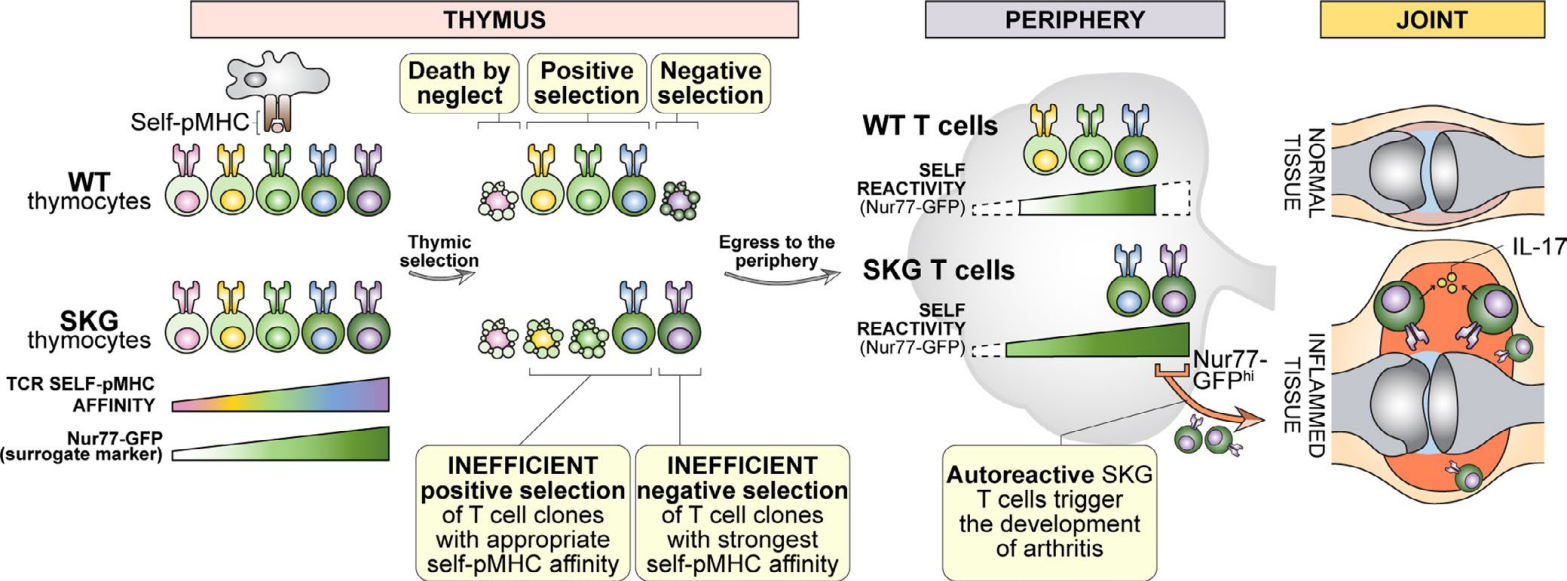
Properties of SKGNur77 GFPhi T cells. Impaired TCR signaling in SKG mice and an altered T cell repertoire along with chronic endogenous antigen encounter confer increased arthritogenicity of selfreactive T cell clones in SKG mice. The most self‐reactive clones (depicted in purple) that have escaped negative selection are marked by the highest levels of Nur77‐eGFP expression (GFPhi). These cells respond more robustly to self‐antigen, are hypersensitive to cytokines like IL‐6, and differentiate more readily into IL‐17 producing T cells. As a consequence, they cause more severe joint tissue damage during early stages of arthritis (Ashouri et al, 2019)

One specific arthritogenic TCR directed against a ubiquitous self‐antigen was identified in the SKG model,^59^ but it was not known whether rare antigen‐specific T cell clones drive disease or whether the entire pre‐immune TCR repertoire has arthritogenic potential because it is highly autoreactive. It is also not clear how tolerance of such clones is broken in the face of profoundly depressed TCR signaling in SKG mice. Therefore, we combined the SKG mice with a fluorescent reporter to read out the relative strength of TCR signaling (termed by us SKGNur mice) to examine antigen‐specific TCR signaling strength in pathologic CD4 T cells.^76^ This reporter tethers GFP expression to the regulatory region of *Nr4a1* (encoding the orphan nuclear hormone receptor Nur77), which is rapidly and selectively upregulated in response to antigen, but not inflammatory stimuli.^77^, ^78^, ^79^ Using the reporter, we identified a subpopulation of CD4 T cells that is greatly enriched for their arthritogenic potential (enriched in the GFP^hi^ relative to GFP^lo^ fractions, see Figure 4).^76^ We found that higher levels of Nur77‐eGFP in SKG CD4 (GFP^hi^) T cells marked their reactivity to self‐pMHC in an autologous mixed lymphocyte reaction (AMLR). We established that these SKG GFP^hi^ naive CD4 T cells were more arthritogenic than the GFP^lo^ subset using an established adoptive transfer model in which SKG CD4 T cells are sufficient and necessary to cause arthritis in a lymphopenic host.^64^, ^76^ The T cells with increased autoreactivity nonetheless had diminished ex vivo‐inducible TCR signaling, perhaps reflective of adaptive inhibitory mechanisms induced by chronic autoantigen exposure in vivo. This enhanced autoreactivity was associated with their ability to more readily differentiate into interleukin‐17 (IL‐17)‐producing cells. Furthermore, the more arthritogenic SKG CD4 T cells were associated with upregulation of IL‐6 cytokine receptor signaling machinery, which might be attributable, in part, to a reduced amount of expression of suppressor of cytokine signaling 3 (SOCS3)—a key negative regulator of IL‐6 receptor signaling. As a result, the more arthritogenic GFP^hi^ CD4 T cells from SKGNur mice were hyperresponsive to IL‐6 receptor signaling.

Similar to our findings in the SKGNur model, analogous antigen‐activated CD4 T cells (enriched for the TCR signaling marker Nur77) were present in RA synovium compared to matched PBMC's from the same donor.^76^ In addition, Nur77 protein expression in CD4 T cells obtained from the RA joints far exceeded that in CD4 T cells isolated from synovial tissue of patients with a nonautoimmune form of arthritis.^76^ These results support the hypothesis that pathogenic CD4 T cells are enriched in RA joints and are likely recognizing an intraarticular autoantigen(s). Consistent with findings from SKGNur mice, *SOCS3* expression was similarly downregulated in RA synovium and suggests a similar mechanism of IL‐6 hyper‐responsiveness in RA joints. Taken together, our findings suggest that despite impaired TCR signaling, autoreactive T cells exposed to chronic antigen stimulation exhibit heightened sensitivity to IL‐6 receptor (IL‐6R) signaling, which contributes to their arthritogenicity in SKG mice (Figure 4), and perhaps in patients with RA.

Recently, we have characterized these arthritogenic SKG CD4 T cells in greater detail. We have performed single‐cell RNA and TCR sequencing analyses and have identified genes that are up‐ or downregulated and have also identified TCRs that are enriched in this subset of pathogenic T cells even prior to the development of arthritis. Our unpublished data suggest that impaired negative selection of of thymocytes with TCRs that recognize endogenous antigens in SKG mice creates a biased repertoire of autoreactive naive CD4 T cells that are poised to contribute to disease pathogenesis through their altered transcriptome as they encounter antigen in the periphery.

## 3 INCREASED ZAP70 FUNCTIONAL EFFECTS ON THE MAINTENANCE OF T CELL TOLERANCE

Recent studies on two hypermorphic ZAP70 mutations, R360P and W131A, illustrate how increased ZAP70 function associated with disrupting ZAP70 autoinhibtion impacts T cell function and immune tolerance (Figure 3).^80^, ^81^, ^82^, ^83^

### 3.1 Weakening ZAP70 autoinhibition results in increased ZAP70 function

While both R360P and W131A mutations destabilize ZAP70 autoinhibition and render the kinase partially constitutively active, structural analyses suggest that the mechanisms of interference are distinct (Figures 2 and 3).^84^, ^85^ W131A disrupts the interdomain A‐interdomain B interaction, which is regulated by phospho‐ITAM binding. In contrast, the R360P mutation appears to relieve the interdomain B and kinase domain interaction (Figures 2 and 3), a role regulated by LCK‐mediated phosphorylation of Y315 and Y319. The activating effects of these two mutations are further supported by biochemical analyses. When we co‐expressed LAT with the full‐length ZAP70 W131A mutant in human embryonic kidney (HEK) 293 cells, we observed a moderate degree of spontaneous LAT phosphorylation.^86^ In contrast, spontaneous LAT phosphorylation was initially not detected in HEK293 cells transfected with the full‐length R360P mutant.^80^ In follow‐up studies when HEK293 cells were transfected with LAT and the ZAP70 R360P mutant lacking the SH2 domains and interdomain A segment, we observed spontaneous LAT phosphorylation by the R360P mutant.^83^ Together, these results support the notion that both the W131A and R360P mutations directly weaken ZAP70 autoinhibition, with the R360P mutant behaving as a weaker hypermorphic allele compared to the stronger W131A hypermorphic mutant allele in TCR signaling.

### 3.2 ZAP70 R360P contributes to human autoimmunity by interfering with peripheral tolerance mechanisms

Notably, the R360P mutant is associated with a recently discovered complex familial autoimmune syndrome characterized by bullous pemphigoid, glomerulonephritis, colitis, and autoantibody to factor VIII.^80^ In this family, two out of three children born to healthy parents were affected by this autoimmune syndrome without evidence of immunodeficiency. Both affected children carried compound heterozygote missense mutations, R192W and R360P, in ZAP70. Structural modeling and biochemical studies revealed that the R192W is a loss‐of‐function allele due to reduced binding of mutant ZAP70 to the phosphorylated ζ‐chain.^80^ Consistent with our findings in HEK293 T cells, transient overexpression of the ZAP70 R360P mutant in ZAP70‐deficient P116 Jurkat cells resulted in increased TCR‐induced signaling and expression of the activation marker CD69. Additionally, the activating effects of the R360P allele were only revealed in the presence of the hypomorphic R192W allele, but were neutralized by wildtype ZAP70.^80^ This is consistent with the clinical observations that both the father and a sister who carry only the R360P heterozygote mutation are healthy. Together, these results suggest that the hypermorphic R360P mutation is most likely responsible for the autoimmunity seen in these patients.

Our recent work on ZAP70 R360P homozygote knock‐in mice provides further insights into the mechanism by which the R360P mutation contributes to the complex autoimmune syndrome in humans.^83^ In the context of a polyclonal TCR repertoire, the R360P mutation has no apparent impact on T cell development. However, we observed a substantial increase in the percentage of central memory CD8 T cells in the lymph nodes of 8‐week‐old R360P mice, suggestive of enhanced TCR tonic signaling in R360P T cells in vivo. Biochemical analyses confirmed that the R360P mutation was a weak hypermorphic allele that increased TCR signaling through weakened ZAP70 autoinhibition, as evidenced in enhanced ZAP70 phosphorylation at Y319 and faster calcium mobilization following anti‐CD3 stimulation.^83^ The impact of R360P on TCR signaling was further revealed when the ovalbumin peptide‐reactive OTI TCR transgene was introduced into the R360P mice, eliminating a possible compensatory TCR repertoire shift. Our utilization of weak peptide agonists for this transgene as TCR stimuli was quite revealing. When CD8 OTI T cells were stimulated by antigen‐presenting cells loaded with a panel of well‐characterized altered chicken ovalbumin peptides (OVA_257‐264_) presented by H‐2K^b^ with varied agonist affinity, the R360P CD8 OTI T cells exhibited selectively enhanced CD69 upregulation and proliferation in response to weak peptide agonists.^83^ Importantly, in the presence of IL‐2, the R360P CD8 OTI T cells exhibited a stronger proliferative response to Catnb,^83^ a naturally occurring, positively selecting self‐peptide for the OTI TCR that is derived from β‐catenin_329‐336_.^87^ Together, our work suggests that the R360P mutation identified in these children contributes to human autoimmunity by selectively allowing increased mature T cell sensitivity to weak and self‐antigens that would otherwise be ignored by T cells with normal ZAP70 function, particularly in the context of a susceptible cytokine milieu or under inflammatory conditions such as following viral infections.

Although ZAP70 is primarily expressed in T cells and NK cells, it is intriguing that these patients with familial autoimmune syndrome developed autoantibody‐mediated diseases including bullous pemphigoid and anti‐factor VIII antibody. Our study of the R360P mice showed that there was an increase in the differentiation of antigen‐specific follicular helper T (Tfh) cells in R360P mice following acute LCMV infection. There were also substantial increases in IgG and IgG1 amounts in 10‐month‐old R360P mice compared with those of control mice, which likely reflects a long‐term consequence of enhanced Tfh cell activity.^83^ These results provide an explanation as to why the R360P mutation in ZAP70 contributes to a break in B cell tolerance in these patients. Thus, as a consequence of enhanced TCR signaling, it is likely that the R360P mutation led to excessive auto‐reactive T helper cell activity for B cells.

Taken together, our work highlights how the R360P mutation contributed to human autoimmunity via impaired autoinhibition of ZAP70 regulation. However, in contrast to human patients, R360P mice do not spontaneously develop a detectable autoimmune phenotype on the C57BL/6 background. This could be multifactorial. It is likely that differences in genetic background, the presence of other genetic modifiers, or unique environmental factors contribute to this discrepancy. Additionally, aged R360P mice accumulate regulatory and anergic T cells,^83^ suggesting compensatory mechanisms such as induction of negative regulatory pathways are engaged to suppress autoimmunity. Future studies on how these negative regulatory mechanisms could be perturbed to break tolerance will enhance our understanding of the pathogenesis of human autoimmune diseases.

### 3.3 The strong hypermorph ZAP70 W131A results in peripheral T cell anergy

Our recent studies on Zap70 W131A mutant mice^81^, ^82^ further illustrate how ZAP70 hypermorphic defects and interference with ZAP70 autoinhibition impact immune tolerance. They reveal the importance of negative regulatory mechanisms in restraining enhanced TCR signaling.

W131A mutant mice with a polyclonal repertoire exhibited relatively normal T cell development and normal TCR‐induced activation suggesting that compensatory mechanisms, such as a TCR repertoire shift, might have masked the effect of the W131A mutation on TCR signaling.^81^ Indeed, introduction of the W131A mutant into the OTII TCR transgenic mice (W131AOTII) resulted in increased negative selection of OTII thymocytes and increased basal TCR signaling, but diminished TCR‐induced signaling.^81^, ^82^ The increased basal TCR signaling revealed by increased calcium, Nur77 levels, and ERK phosphorylation in unstimulated W131AOTII T cells confirmed that W131A is a hypermorphic mutant.^81^, ^82^ The increased, but incomplete, negative selection characterized by 3‐fold increases in frequencies of activated caspase 3+ thymocytes likely resulted from apoptosis as a consequence of OTII TCR recognition in response to an unknown self‐pMHC encountered in the thymus.^82^ Thus, negative selection was a partial but incomplete means of controlling cells with increased TCR signaling due to the W131A mutation and the OTII TCR.

Since not all self‐antigens are effectively presented in the thymus and negative selection mechanisms are likely to be incomplete,^8^ compensatory peripheral tolerance mechanisms may be employed. Such mechanisms may include increased frequencies of anergic cells and Tregs. Indeed, high numbers of peripheral CD4 Tregs and anergic T cells, characterized by their Foxp3^−^ (non‐Treg) CD73^+^FR4^+^ phenotype,^88^ were observed in W131AOTII mice. Anergy induction not only prevents the responsiveness of potentially harmful self‐reactive CD4 T cells but also generates progenitor cells for peripheral Tregs.^88^, ^89^ Tregs actively suppress cytokine production and the proliferation of other immune cells through a variety of mechanisms.^90^ Thus, the strong induction of anergic T cells and Tregs in peripheral W131AOTII mice are tolerance mechanisms that appear to have been employed to keep potentially autoreactive T cells that escaped central tolerance in check.

Peripheral W131AOTII CD4 T cells displayed classical characteristics of T cell anergy, such as greatly impaired TCR‐induced signaling as well as reduced production of IL‐2, proliferation, and induction of activation markers, including CD25, CD69, and Nur77 in response to OVA_323‐339_ peptide or TCR stimulation.^81^, ^82^ Moreover, RNA sequencing data revealed peripheral W131AOTII CD4 T cells demonstrate significantly increased expression of Treg, anergy‐related, and inhibitory gene expression profiles capable of influencing cytokine and TCR signaling.^82^ Thus, the W131AOTII mouse model is a distinctly unique genetic model in which T cell anergy naturally developed without adoptive transfer of T cells or introducing antigens into these mice. The induction of T cell anergy in W131AOTII mice due to encounter with an unidentified self‐pMHC likely mimics the events that normally occur with higher affinity interactions involving self‐pMHC and some clonally distributed TCRs on T cells of normal mice. Future studies will aim to identify the self‐antigens that induce anergic T cells in W131AOTII mice.

We utilized this genetically encoded mouse model of T cell anergy to answer a key question: Is T cell anergy induced and established in the thymus or in the periphery? Using the W131AOTII model, we found that T cell anergy is induced and established in the periphery.^82^ W131AOTII single‐positive CD4 thymocytes that escaped deletion exhibited normal TCR signaling in response to OVA_323‐339_ peptide and TCR stimulation despite evidence of the presence of a relevant self pMHC based on the increased negative selection in the thymus. Consistent with this notion, and in contrast to enriched tolerized gene signatures and impaired TCR signaling observed in peripheral CD4 T cells, CD4 single‐positive thymocytes exhibited biochemical TCR signaling and gene expression in W131AOTII mice that were comparable to those observed in control OTII mice. Therefore, since increased Tregs and anergic T cells were detected in the periphery, our results suggest that peripheral tolerance is not established in the thymus but, instead, is established in the periphery. This likely involves encounter of a self‐ pMHC in the periphery since we found the maintenance of CD4 T cell anergy and survival of anergic T cells in W131AOTII mice required antigen presentation via MHC‐II molecules.

In mice, recent thymic emigrants take up to 3 weeks to differentiate into mature T cells.^91^, ^92^ Consistent with this, we found that 3‐week‐old W131AOTII mice showed small increases in peripheral anergic T cells in contrast to large increases in the frequencies of anergic T cells in adult 8‐week‐old W131AOTII mice.^82^ Since W131AOTII CD4 single‐positive thymocytes are not anergic, recent thymic emigrants in W131AOTII mice might take at least 3 weeks to become mature anergic cells. Future studies will be needed to reveal when and where in the periphery anergic T cells are induced in W131AOTII mice.

Multiple negative regulators that target TCR signaling pathways, including PD‐1 and E3 ubiquitin ligases, are highly expressed in anergic T cells and contribute to establishing the unresponsive state in anergic T cells.^93^, ^94^, ^95^ PD‐1, an ITIM containing inhibitory receptor, inhibits T cell responses by recruiting the SH2 domain‐containing phosphatase SHP2 which targets protein tyrosine kinase‐dependent TCR and co‐stimulatory CD28 pathways.^96^, ^97^ The E3 ubiquitin‐protein ligases Cbl‐b and Grail recognize and transfer ubiquitin from specific E2 ubiquitin‐conjugating enzymes to target substrates, promoting substrate degradation by the proteasome or lysosome.^98^ Cbl‐b interacts with and inhibits multiple downstream molecules of protein tyrosine kinase‐dependent TCR and CD28 pathways, such as PI3K, LCK, ZAP70, Vav, PLC‐γ, PKC‐δ, Grb2, and others.^99^, ^100^ Grail is also a negative regulator of TCR responsiveness and cytokine production by targeting the TCR ζ chain and the IL‐21 receptor.^101^, ^102^, ^103^, ^104^ Previous studies suggested that deletion of Cbl‐b or Grail in T cells results in hyper‐responsiveness to TCR stimulation and resistance to anergy induction.^100^, ^102^ Furthermore, W131AOTII T cells showed increased protein expression of PD‐1 and Cbl‐b.^81^, ^82^ Thus, PD‐1, Cbl‐b and Grail were candidate mediators of T cell unresponsiveness in the anergic state.

We examined the relative contribution of these three negative regulators in the W131AOTII model using mice deficient in each of these negative regulators.^82^ Interestingly, loss of Cbl‐b, Grail, or PD‐1 did not affect the numbers of phenotypically identified anergic CD4 T cells or numbers of Treg in W131AOTII mice. However, deletion of Cbl‐b, but not Grail or PD‐1, in W131AOTII mice restored T cell responsiveness and signaling to OVA peptide or TCR‐induced stimulation. Consistent with the restoration of calcium‐dependent responses, we found IL‐2 production in Cbl‐b^−/−^W131AOTII T cells relied on NFAT‐dependent signaling pathways, based on sensitivity to cyclosporin A. Furthermore, loss of Cbl‐b in W131AOTII mice resulted in profound differences in the gene expression profiles associated with improved T cell responsiveness.^82^ Thus, Cbl‐b appears to play an essential role in the establishment and/or maintenance of unresponsiveness in T cell anergy. Moreover, our studies suggest that cell surface markers associated with the anergic state may not be reliable markers of T cell unresponsiveness. How Cbl‐b deficiency prevents the function, but not the phenotype in W131AOTII anergic T cells deserves further investigation to understand this phenotypic/functional dissociation.

Although germline PD‐1 deletion did not rescue functional anergy, we had previously found that PD‐1 blockade by anti‐PD‐1 treatment partially eliminated T cell unresponsiveness in young 4‐week‐old W131AOTII mice.^81^ It is not clear, from our studies, whether anti‐PD‐1 prevented the establishment of anergic T cells in newly developed T cells or whether it reversed unresponsiveness in a subset of previously established anergic T cells. Grail^−/−^ and PD‐1^−/−^ T cells were reported to be resistant to other anergic models, including in vitro culture with ionomycin or TGF‐β or Treg, or in vivo oral tolerance or peptide‐induced anergy.^105^, ^106^, ^107^, ^108^ However, in our studies germline deletion of PD‐1 or Grail in W131AOTII mice did not prevent the development of anergic T cells or their unresponsiveness.^82^ Therefore, there may be developmentally layered compensatory mechanisms in PD‐1 or Grail germline deleted mice in order to maintain the anergic state in W131AOTII T cells or different modes of anergy induction may involve distinct mechanisms. Thus, these results suggest that many layers of compensatory mechanisms control T cell anergy and maintain peripheral tolerance.

Taken together, our studies with ZAP70 hypermorphic mutants suggest that both central and peripheral tolerance mechanisms may be engaged in systems for which heightened TCR signaling must be compensated. Our genetically encoded mouse model of T cell anergy reveals that T cell anergy is induced in the periphery. The maintenance of the anergized unresponsive state is dependent on Cbl‐b, but not PD1 or Grail. Understanding the mechanisms governing T cell unresponsiveness in anergic T cells may contribute to the development of new therapeutic strategies to induce tolerance in the setting of autoimmune diseases.

### 3.4 Altered ZAP70‐dependent downstream signaling exhibits similar effects on immunodeficiency and autoimmunity in humans

**FIGURE 5:**
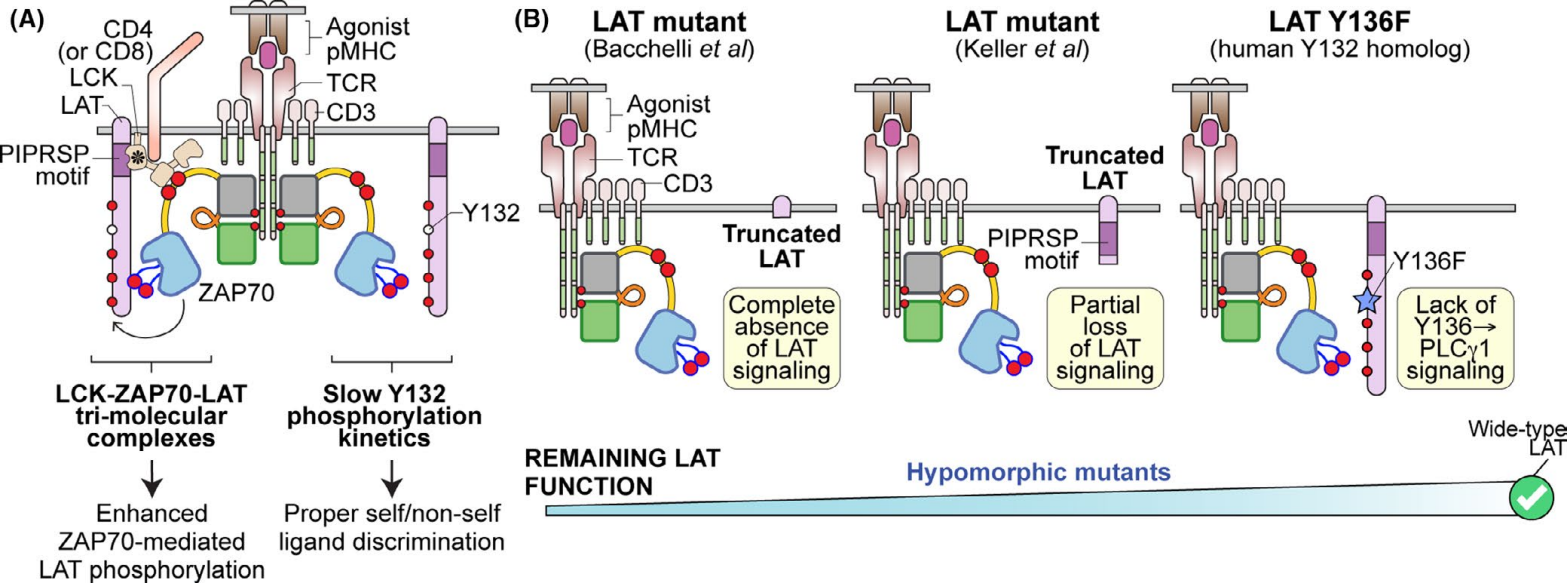
Mutation of LAT. (A) Upon recognition of an agonist MHC, LAT is phosphorylated by active ZAP70 on LAT's five key tyrosine residues. The phosphorylation kinetics of LAT plays an essential role in determining TCR sensitivity and ligand discrimination. First, LCK may bridge active ZAP70 with LAT to facilitate ZAP70‐mediated LAT phosphorylation. This tri‐molecular complex (LCK‐ZAP70‐LAT) enhances T cell sensitivity toward weak ligand. Secondly, the slow phosphorylation of LAT Y132 (human) or Y136 (mouse) is a key signaling bottleneck to ensure that only a bona‐fide activating signal can trigger T cell activation. (B) Hypomorphic LAT mutants and the resulting signaling effects are illustrated

As discussed above, ZAP70 activation is a key step prior to the diversification of TCR signal transduction pathways that elicit T cell activation.^16^ Activated ZAP70 phosphorylates the transmembrane adaptor LAT on five key tyrosine residues: Y127, Y132, Y171, Y191, and Y226 (annotated based on human LAT amino acid sequences, see Figures 1B and 5).^109^ After being phosphorylated, these tyrosine residues in LAT serve as docking sites to recruit other enzymes (eg, Y132 for PLCγ1) or adaptor molecules (eg, Y171 and Y226 for Grb2; Y171 and Y191 for GADS), in turn nucleating the assembly of “LAT signalosomes”. LAT signalosomes are regarded as signaling hubs for TCR signal divergence. Thus, one major task of activated ZAP70 is to initiate LAT signaling in order to bridge antigen receptor triggering to the downstream signals required for full T cell activation, including calcium increases and PKC and Ras‐MAPK activation, among others.^6^, ^109^

Recently, we and others have discovered that this signaling step—ZAP70‐mediated LAT phosphorylation—plays a critical role in determining T cell sensitivity and ligand discrimination. The efficiency and kinetics of LAT phosphorylation not only allow TCR signals to propagate and LAT signalosomes to assemble but also through a specific spatial and temporal sequence to orchestrate the dynamics of signaling proteins.^28^, ^34^, ^109^, ^110^, ^111^

We recently found that phosphorylation of LAT is spatially regulated within the vicinity of the agonist pMHC‐engaged TCR complexes through the formation of LCK‐ZAP70‐LAT tri‐molecular complexes following TCR stimulation.^28^, ^34^, ^109^ This spatial arrangement allows ZAP70‐mediated phosphorylation of LAT to occur efficiently, especially in the context of weak agonist stimulation. This is driven by the adaptor functions of LCK. On the one hand, LCK is a kinase whose role is to phosphorylate ITAMS and as well as to recruit and activate ZAP70 to the engaged TCR complexes. On the other hand, LCK also functions as an adaptor to connect LAT (ZAP70 substrate) with activated ZAP70. Using evolutionary conservation analysis and site‐directed mutagenesis together with mass spectroscopy proteome interaction analysis, our work identified that LCK utilizes its SH3 domain to actively recruit LAT via LAT's proline‐rich motif PIPRSP to the engaged TCR complexes.^28^ Combined with LCK's co‐receptor association, this tri‐molecular complex plays an important role in supporting the signaling from the pMHC‐engaged TCR complexes. Indeed, stimulating T cells with antigen‐loaded tetramers successfully revealed the LCK‐ZAP70‐LAT multiple protein complexes in the initiation phase of TCR signaling.^28^ Computational analysis and T cell activation assays further provide mechanistic insights that this signaling step augments TCR sensitivity to weak ligands.^28^, ^34^ The importance of the LCK SH3 domain has been previously shown to contribute to TCR downstream signaling including LAT phosphorylation and is consistent with our observations.^112^ Taken together, these data support that there is a spatial regulatory mechanism embedded in ZAP70‐involved signaling to modulate TCR signaling initiation.

Interestingly, the public database for human single‐nucleotide variations shows a naturally occurring missense SNP (dsSNP reference cluster id: rs41292396) localized within that specific PIPRSP motif in LAT that is required for the association of Lck‐ZAP70‐LAT multiple protein complexes. Ectopic expression of this SNP‐encoded LAT variant shows impaired TCR signaling (unpublished data), similar to signaling defects observed in T cells that express the mutant LAT that lacks the functional PIPRSP motif.^28^ Data mined from various databases indicate a very rare frequency of this SNP in western populations. Future studies may be warranted to explore whether this SNP rs41292396 could be associated with increased risk for the development of autoimmune disease or immunodeficiency.

Two recent studies have offered clinical insights into *LAT* genetic mutations in humans.^113^, ^114^, ^115^ In one study by Bacchelli et al,^115^ a homozygous frameshift mutation in the gene encoding LAT was identified in five patients from a single consanguineous pedigree. The frameshift mutation produced a mutant LAT protein truncated shortly after the transmembrane domain (Figure 5).^115^ The affected individuals had extremely low numbers of CD4 and CD8 T cells (10‐210 cells/mm^3^ and 0‐30 cells/mm^3^, respectively), consistent with impaired T cell development observed in *Lat*‐deficient murine models,^70^, ^116^, ^117^ while the B cell and natural killer cell populations were relatively unaffected.^115^ This virtual absence of a functional T cell compartment caused all five patients to suffer from recurrent infections and severe combined immunodeficiency (SCID). Ectopic expression of the patient's mutant LAT in LAT‐deficient Jurkat cells confirmed the inability of the truncated LAT to support proximal TCR signaling.^115^

Interestingly, similar to the hypomorphic ZAP70 mutations that can lead to immunodeficiency and/or autoimmunity in humans,^37^, ^40^, ^64^, ^67^, ^76^ a second study of *LAT* mutations presents a more complicated phenotype with some unexpected clinical and immunologic features.^113^, ^114^ Keller et al,^113^ evaluated three patients from a single family that all harbor a homozygous mutation in the *LAT* genomic locus that also resulted in a C‐terminal partially truncated LAT protein. However, this mutant LAT has a short intracellular tail (the first 88 amino acids) which lacks all five key tyrosine residues (Figure 5). Similar to the first study, this truncated LAT mutant was unable to restore calcium mobilization and T cell activation in LAT‐deficient Jurkat variant JCam2 cells. Despite this, characterization of these patients' primary T cells exhibited heterogenous phenotypes.^113^ One patient's T cells were still capable of mediating calcium responses, albeit delayed, with comparable NFκB signaling.^113^ In some patients, the *LAT* mutation resulted in immunodeficiency, while in others it led to autoimmune disease.^113^ We posit that the dysregulated TCR signaling due to the LAT mutations can result in a spectrum of disease phenotypes from immune deficiency to autoimmunity depending on compensatory signaling mechanisms and additional environmental triggers. Notably, a higher percentage of patients' T cells spontaneously produced cytokine IL‐4 and demonstrated strong T helper 2 (Th2) biased phenotypes.^113^ This finding was surprisingly similar to a mutant mouse line in which the Y136 residue was substituted with a phenylalanine (LAT‐Y136F mice; murine Y136 corresponds to human Y132, Figure 5).^70^, ^118^, ^119^, ^120^, ^121^ The Y136F mutation abrogated calcium mobilization, PLCγ1 signals, and NFAT signals in the mutant T cells, thereby disrupting normal T cell thymic development. Nonetheless, the lymphopenia conditions in Y136F mice could result in Th2‐biased lymphoproliferation, spontaneous follicular helper T cell development, and autoimmune nephritis and fibrosis.^69^, ^70^, ^118^, ^122^, ^123^, ^124^

Several possible mechanisms may explain how impaired TCR signaling leads to such autoimmune‐like phenotypes were discussed earlier in this review. Here we will discuss how LAT's role in TCR signaling may further contribute to the various observed clinical phenotypes. First, a key difference between the two clinical cases is the remaining intracellular domain of LAT.^113^, ^115^ Bacchelli et al,^113^, ^115^ identified a mutant LAT which essentially completely lacks the intracellular domain of LAT, whereas the mutant LAT characterized by Keller et al,^28^, ^109^ still retains the N‐terminal membrane‐proximal region. The significance of LAT's membrane‐proximal intracellular region was previously unappreciated. However, we discovered that this membrane‐proximal region of LAT possesses highly evolutionarily conserved proline sequences. As discussed above, we showed that the PIPRSP motif in this region can interact with the co‐receptor‐associated LCK SH3 domain to form a higher order of tri‐molecular complexes with ZAP70.^113^, ^115^ Moreover, in addition to the PIPRSP motif, there are additional PAYPP and PWPP motifs that could also potentially possess some signaling functions that are not yet defined. These three proline‐rich motifs located in the membrane‐proximal regions that we identified are all still intact in the patients studied by Keller et al^113^, ^114^ Thus, it is possible that the mutant LAT described by Keller et al,^125^, ^126^, ^127^ may have some residual function. Second, it is feasible that compensatory signals may have been adopted by the affected individuals' mutant T cells resulting in divergent clinical phenotypes. One potential candidate is CD6. A recent study by Mori and colleagues took advantage of mass spectrometry to profile the coordinated signaling dynamics between the CD6 signalosome and the LAT signalosome.^126^ Interestingly, while LAT signalosomes were determined to play a positive role in TCR signaling, the CD6 signalosome can be either inhibitory or stimulatory, dependent on the relative signaling strength of LAT signalosome. Specifically, the loss of LAT signalosomes may likely shift the signaling characteristics of CD6 as an inhibitory receptor to a stimulatory receptor, raising the possibilities of inducing downstream TCR signals in the absence of LAT. Therefore, although Keller et al,^113^, ^114^ did not observe altered CD6 expression levels in the patients' T cells, it is possible that the signaling role of CD6 has been shifted from an inhibitory role to a positive one in the absence of LAT signals.

Additionally, considering the similarities between the Y136F murine^70^, ^118^, ^119^, ^120^, ^121^ and the partially truncated *LAT* caused autoimmune phenotypes in patients,^113^ one could speculate that the analogous human and murine tyrosines in LAT that bind PLCγ1 may promote some similar essential signaling function in controlling T cell quiescence. We recently showed that the phosphorylation kinetics of Y136/Y132 play a critical role in supporting T cell self and non‐self‐ligand discrimination.^110^ Specifically, the Y132 phosphorylation rate is the slowest one among all five key tyrosine residues. This uniquely slow phosphorylation of Y132 in LAT is due to the neutral amino acid (glycine) in the −1 position. The ZAP70 kinase domain substrate‐binding region is heavily charged, and therefore ZAP70 usually prefers a negatively charged amino acid (glutamic acid or aspartic acid) N‐terminal to a substrate tyrosine. Indeed, all ZAP70 tyrosine substrates have a negatively charged amino acid as the preceding residue, except for the glycine preceding Y132 in human LAT, or all its corresponding residues in tetrapod T cells, implying a highly evolutionarily conserved feature. Interestingly, we discovered that some fish, including zebrafish, have a negatively charged residue prior to Y132/Y136 LAT homolog, which leads to much faster phosphorylation kinetics of this specific tyrosine in LAT in fish T cells. This unusually rapid kinetics of Y132 phosphorylation in the fish LAT homolog seems able to support fish T cells to promote LAT‐PLCγ1‐calcium responses in a cold environment (~13°C), whereas mammalian T cells, including human and mouse, were unable to do so.^110^ Ectopic expression of the fish version of LAT may confer mouse primary T cells with similar capacities: a faster phosphorylation rate of Y132/Y136 endows them with a more robust and faster PLCγ1‐calcium response. These “engineered” murine T cells that have an acidic residue N‐terminal to Y136 have a higher sensitivity toward self‐peptides or weak ligands that normally were not able to activate wildtype T cells.^110^ In other words, LAT Y132/Y136‐calcium‐PLCγ1 signaling is essential to support T cells with proper self and non‐self‐ligand discrimination, and sequence engineering the amino acid prior to LAT Y132/Y136 may modulate T cell responses and sensitivity. Interestingly, this tyrosine Y132/Y136 in LAT is the only tyrosine residue in LAT that, upon phosphorylation, can recruit and activate PLCγ1, eventually leading to calcium mobilization and NFAT signaling. Despite this, human T cells with the LAT Y132 mutation were still able to induce some degree of calcium responses.^113^ This unexpected finding, along with the apparent role of LAT Y132/Y136 in TCR ligand discrimination, raises the possibility for future studies to elucidate how LAT‐dependent calcium responses may differ from those of LAT‐independent calcium responses to modulate T cell sensitivity and to control cell fate decisions between T cell activation or quiescence.

## 4 CONCLUSION

As one of the first kinases to be activated during TCR activation, ZAP70 plays a central role in orchestrating T cell functionality. Gained from the insights of studying *ZAP70* mutations in mouse and human, our laboratory has established a panel of ZAP70 variant alleles that exhibit a wide spectrum of hyporeactive or hyperreactive ZAP70 activities. Interestingly, both hypoactive and hyperactive ZAP70 may lead to the development of autoimmune diseases, albeit through distinct mechanisms. Immature thymocytes, and mature T cells rely on normal ZAP70 function to complete their developmental process in the thymus or to modulate T cell responses in the periphery. Hypoactive ZAP70 activity compromises the developmental checkpoints that are important to maintain central tolerance, allowing thymocytes with potentially self‐reactive TCRs to have a greater chance to escape negative selection. Such “forbidden clones” may escape into the periphery, and eventually pose a greater risk to cause autoimmune disease. In fact, the majority of mutations in *ZAP70* that are associated with immunodeficiency or autoimmunity in human and mice are hypomorphic alleles that result in complete or partial loss of ZAP70 function.^37^, ^40^, ^64^, ^67^, ^76^ On the other hand, hyperreactive ZAP70 may enhance thymic negative selection and but endow T cells that escape negative selection with greater capacities to engage peripheral tolerance mechanisms, including selectively enhancing T cell sensitivity to weak and self‐ligands, the induction of anergy, expansion of Tregs, and upregulation of inhibitory receptors or signaling molecules. Consistent with the findings in *Zap70* mutants, the signaling defects in ZAP70 substrate, LAT, can also lead to autoimmune diseases. Taken together, our work and others reveal the essential role of ZAP70 in maintaining central tolerance and peripheral tolerance. Considering that each *Zap70* mutant we have studied may abrogate a specific function or regulation of ZAP70, it is likely that other mutations that disturb TCR signaling thresholds may influence tolerance mechanisms and cause autoimmunity. Future work will aim to further dissect the similarities and differences among mouse models of patient disease or mutations in *ZAP70* that affect TCR signaling. The resulting insights will help us delineate the role of each regulatory step of ZAP70 activity in determining T cell functions and responses.

## CONFLICTS OF INTEREST

All authors declare they have no conflicts of interest to disclose.

## Data Availability

All presented data have been published and are available upon request.
